# Membrane-bound *sn*-1,2-diacylglycerols explain the dissociation of hepatic insulin resistance from hepatic steatosis in MTTP knockout mice

**DOI:** 10.1194/jlr.RA119000586

**Published:** 2020-09-09

**Authors:** Abudukadier Abulizi, Daniel F. Vatner, Zhang Ye, Yongliang Wang, Joao-Paulo Camporez, Dongyan Zhang, Mario Kahn, Kun Lyu, Alaa Sirwi, Gary W. Cline, M. Mahmood Hussain, Patricia Aspichueta, Varman T. Samuel, Gerald I. Shulman

**Affiliations:** 1 Department of Internal Medicine, Yale School of Medicine, New Haven, CT, USA; 2 Departments of Cell Biology and Pediatrics, SUNY Downstate Medical Center, Mineola, NY, USA; 3 Department of Foundations of Medicine, NYU Long Island School of Medicine, Mineola, NY, USA; 4 Department of Physiology, Faculty of Medicine and Nursing, University of the Basque Country UPV/EHU, Leioa, Spain; 5 Biocruces Research Institute, Barakaldo, Spain; 6 Veterans Affairs Medical Center, West Haven, CT, USA; 7 Department of Cellular and Molecular Physiology, Yale School of Medicine, New Haven, CT, USA

**Keywords:** lipids, liver, metabolic disease, nonalcoholic fatty liver disease, drug therapy, liver microsomal triglyceride transfer protein, diabetes, liver-targeted mitochondrial uncoupler

## Abstract

Microsomal triglyceride transfer protein (MTTP) deficiency results in a syndrome of hypolipidemia and accelerated NAFLD. Animal models of decreased hepatic MTTP activity have revealed an unexplained dissociation between hepatic steatosis and hepatic insulin resistance. Here, we performed comprehensive metabolic phenotyping of liver-specific MTTP knockout (L-*Mttp*^−/−^) mice and age-weight matched wild-type control mice. Young (10–12-week-old) L-*Mttp*^−/−^ mice exhibited hepatic steatosis and increased DAG content; however, the increase in hepatic DAG content was partitioned to the lipid droplet and was not increased in the plasma membrane. Young L-*Mttp*^−/−^ mice also manifested normal hepatic insulin sensitivity, as assessed by hyperinsulinemic-euglycemic clamps, no PKCε activation, and normal hepatic insulin signaling from the insulin receptor through AKT Ser/Thr kinase. In contrast, aged (10-month-old) L-*Mttp*^−/−^ mice exhibited glucose intolerance and hepatic insulin resistance along with an increase in hepatic plasma membrane *sn*-1,2-DAG content and PKCε activation. Treatment with a functionally liver-targeted mitochondrial uncoupler protected the aged L-*Mttp*^−/−^ mice against the development of hepatic steatosis, increased plasma membrane *sn*-1,2-DAG content, PKCε activation, and hepatic insulin resistance. Furthermore, increased hepatic insulin sensitivity in the aged controlled-release mitochondrial protonophore-treated L-*Mttp*^−/−^ mice was not associated with any reductions in hepatic ceramide content. Taken together, these data demonstrate that differences in the intracellular compartmentation of *sn*-1,2-DAGs in the lipid droplet versus plasma membrane explains the dissociation of NAFLD/lipid-induced hepatic insulin resistance in young L-*Mttp*^−/−^ mice as well as the development of lipid-induced hepatic insulin resistance in aged L-*Mttp*^−/−^ mice.

Microsomal triglyceride transfer protein (MTTP) plays a critical role in the assembly and secretion of VLDL in the liver and chylomicrons in the intestine (1–3). MTTP deficiency results in abetalipoproteinemia, a rare genetic disorder resulting in reduced plasma APOB containing lipoproteins and increased NAFLD (4). The pharmacological inhibition of MTTP is currently being used to treat patients with homozygous familial hypercholesterolemia, helping to significantly reduce LDL-C- and APOB-containing lipoprotein levels in these patients (5, 6). Of concern, pharmacologic MTTP inhibition is associated with significant toxicities, including hepatic steatosis and increased liver aminotransferase levels (7).

In mice, the genetic deletion of liver MTTP induces hepatic fat accumulation; however, despite hepatic steatosis, these mice demonstrated normal hepatic insulin sensitivity (1, 8). In fact, chronic treatment with an MTTP inhibitor apparently improved glucose tolerance in Zucker rats (9). The metabolically neutral phenotype contrasts with the well-established relationship between hepatic lipid accumulation and hepatic insulin resistance in most humans with NAFLD and the majority of rodent models of NAFLD (10). The mechanism responsible for lipid-induced hepatic insulin resistance has been attributed to hepatic DAG activating PKCε, which promotes insulin receptor kinase threonine^1160^ (mouse threonine^1150^) phosphorylation, thereby inhibiting insulin-stimulated insulin receptor kinase tyrosine phosphorylation (11). There are important exceptions to the relationship between hepatic steatosis and hepatic insulin resistance in rodents, such as when excess hepatic lipid is limited to metabolically neutral subcellular compartments (12). Thus, while MTTP inhibition clearly lowers plasma lipids and increases hepatic lipids, it is unclear how this hepatic steatosis is associated with or dissociated from a metabolically deleterious phenotype.

To better understand the effects of MTTP ablation over time, we studied both young and aged liver-specific MTTP knockout mice (L-*Mttp*^−/−^), assessing the development of lipid-induced hepatic insulin resistance. Furthermore, we assessed the content and subcellular localization of different stereochemical isoforms of DAG in the liver of L-*Mttp*^−/−^ mice. In addition, we assessed the utility of a liver-targeted mitochondrial uncoupling agent (13–17) for the treatment of hepatic steatosis in L-*Mttp*^−/−^ mice, as there are no currently available treatments for hepatic steatosis in the setting of MTTP deficiency. The orally bioavailable functionally liver-targeted controlled-release formulation of 2,4-dinitrophenol (controlled-release mitochondrial protonophore; CRMP) reverses insulin resistance, hypertriglyceridemia, NASH, and diabetes in rodent and nonhuman primate models of T2D and NAFLD/NASH (14–17). Therefore, after assessing the relationship between MTTP ablation and lipid-induced hepatic insulin resistance, we administered CRMP to evaluate the potential value of liver-targeted mitochondrial uncoupling for the treatment of hepatic steatosis and hepatic insulin resistance due to reduce MTTP activity.

## MATERIALS AND METHODS

### Animal care

All experimental procedures were approved by and conducted in accordance with the Institutional Animal Care and Use Committee at Yale University. Liver-specific MTTP knockout mice (L-*Mttp*^−/−^) from a C57BL/6 background were generated as described previously (18, 19). In all studies, age- and weight-matched WT (*Mttp^f/f^*) mice served as controls. Mice were individually housed under controlled temperature (∼23°C) and lighting (12-h light/dark cycle, lights on at 7:00 AM) with free access to water and food. Mice were maintained with regular chow (Envigo 2108S; 24% protein/58% carbohydrates/18% fat calories). Mice were fasted overnight for infusion studies and 6 h for basal measurements. Body composition was assessed by ^1^H magnetic resonance spectroscopy using a Bruker BioSpin Minispec analyzer. Energy expenditure, respiratory quotient, oxygen consumption (Vo_2_), carbon dioxide production (Vco_2_), locomotor activity, and food intake were measured using a comprehensive laboratory animal metabolic system (CLAMS; Columbus Instruments). Drinking in the metabolic cages was measured as described previously (20). CRMP was mixed in peanut butter (2 mg/kg) and administered orally; control animals in the CRMP treatment experiment received peanut butter plus vehicle. In this study, young mice were 10–12 weeks old, whereas aged mice were 10 months old.

### Glucose tolerance test

Following an overnight fast, mice were injected intraperitoneally with 1 g/kg dextrose. Blood samples were taken by tail massage for glucose and insulin measurements at 0, 15, 30, 60, and 120 min.

### Hyperinsulinemic-euglycemic clamp

Hyperinsulinemic-euglycemic clamps were performed in conscious mice as previously described (21). [3-^3^H]glucose (PerkinElmer) was infused at a rate of 0.05 μCi/min for 120 min to assess basal turnover. Following the basal infusion, human insulin (Novo Nordisk) was given as a prime [7.14 mU/(kg-min) × 3 min] and then continuous [2.5 mU/(kg-min)] infusion along with a variable infusion of 20% dextrose to maintain euglycemia (100–120 mg/dL) and [3-^3^H]glucose at a rate of 0.1 μCi/min. Plasma samples were obtained by tail massage at 0, 25, 45, 65, 80, 90, 100, 110, 120, 130, and 140 min. At the end of the study, mice were euthanized with a sodium pentobarbital injection (∼4 mg/mouse), and tissues taken were snap-frozen in liquid nitrogen and stored at −80°C for subsequent use.

### Plasma assays

Plasma glucose was measured using a YSI 2700D glucose analyzer (Yellow Springs Instruments). Standard kits were used to measure plasma nonesterified fatty acids (Wako) and triglycerides (Sekisui). Insulin concentrations were determined by radioimmunoassay (EMD Millipore).

### Liver lipid measurements

Tissue triglycerides (TAGs) were extracted using the method of Bligh and Dyer (22) and measured using a standard kit (Sekisui). For subcellular compartment-specific DAG extraction, liver tissue was homogenized with a Doucne-type homogenizer in Buffer A [250 mM sucrose, 10 mM Tris (pH 7.4), 0.5 mM EDTA]. The full extraction was performed on ice or at 4°C. The homogenate was centrifuged at 17 K rcf for 15 min to obtain supernatant 1 and pellet 1. Supernatant 1 was centrifuged at 387 K rcf for 75 min; the resultant pellet contained ER membrane lipids, the top layer of the supernatant contained lipid droplet lipids, and the middle of the supernatant contained cytosolic lipids. Pellet 1 was resuspended in Buffer A, layered on top of a 1.12 M sucrose solution, and centrifuged at 111 K rcf for 20 min to obtain pellet 2 and supernatant 2. Pellet 2 was resuspended in Buffer A and centrifuged at 17 K rcf for 15 min; the resultant pellet contained mitochondrial membrane lipids. The interfacial layer of supernatant 2 was taken and diluted with Buffer A and centrifuged at 59 K rcf for 9 min; the resultant pellet contained plasma membrane lipids.

The separation and quantitation of DAG stereoisomers were performed by LC-MS/MS using electrospray ionization on an AB Sciex Qtrap 6500 interfaced to a Shimadzu UFLC using Luna 5u Silica (100 Å, 250 × 2.0 mm) and LUX 5u Cellulose-1 (250 × 4.6 mm) columns connected in series with an isocratic solvent of hexane-isoproponal (300:7). DAG stereoisomer standards were used to establish retention times and responses relative to the internal standard (C17, C17-DAG). No additional separation following subcellular fractionation was done prior to LC-MS/MS analysis to avoid racemization. DAG content is expressed as the sum of individual species. Hepatic ceramide content was measured as previously described (23).

### Immunoblotting analysis

Tissue was homogenized in ice-cold homogenization buffer with protease and phosphatase inhibitors (Complete MINI + PhosSTOP; Roche). Protein extracts (30 µg) were separated by a 4%–12% gradient SDS-PAGE (Invitrogen) and then transferred to a PVDF membrane (Millipore) using a semidry transfer cell (Bio-Rad) for 2 h. After blocking with 5% nonfat dry milk in TBST [10 mM Tris (pH 7), 100 mM NaCl, 0.1% Tween 20], membranes were incubated overnight at 4°C with primary antibodies. Membranes were thoroughly washed and incubated with the appropriate secondary antibody (Cell Signaling Technology), and immune complexes were detected using an enhanced luminol chemiluminescence system (Thermo Fisher Scientific) and exposed to photographic film. Immunoblots were quantified by optical densitometry. For PKCε translocation, cytoplasm and plasma membrane were separated by ultracentrifugation as previously described (24, 25) prior to Western blotting. Insulin receptor β, phosphorylated insulin receptor β, Akt, phosphorylated Akt (Ser473), IRE1, phosphorylated eiF2, and GAPDH antibodies were purchased from Cell Signaling Technology. Sodium potassium ATPase and phosphorylated IRE1 antibodies were purchased from Abcam Inc. PKCε antibody was purchased from BD Biosciences. Antibody against eiF2 was purchased from Santa Cruz Biotechnology. KDEL (GRP 78 and GRP 94) antibody was purchased from Enzo Life Science.

### Markers of liver inflammation

Inflammatory cytokines were measured in liver homogenates by ELISA (Qiagen) and normalized to total protein content by a standard Coomassie-based absorption assay (Thermo Fisher Scientific).

### Statistical analysis

All data are expressed as means ± SEMs. Results were assessed using a two-tailed unpaired Student *t*-test or two-way ANOVA followed by Tukey’s multiple comparison test (Prism 7; GraphPad Software, Inc.). *P* < 0.05 was considered significant.

## RESULTS

### Young L-*Mttp*^−/− ^mice exhibit hepatic steatosis without excess *sn*-1,2-DAG at the plasma membrane

**Fig. 1.**
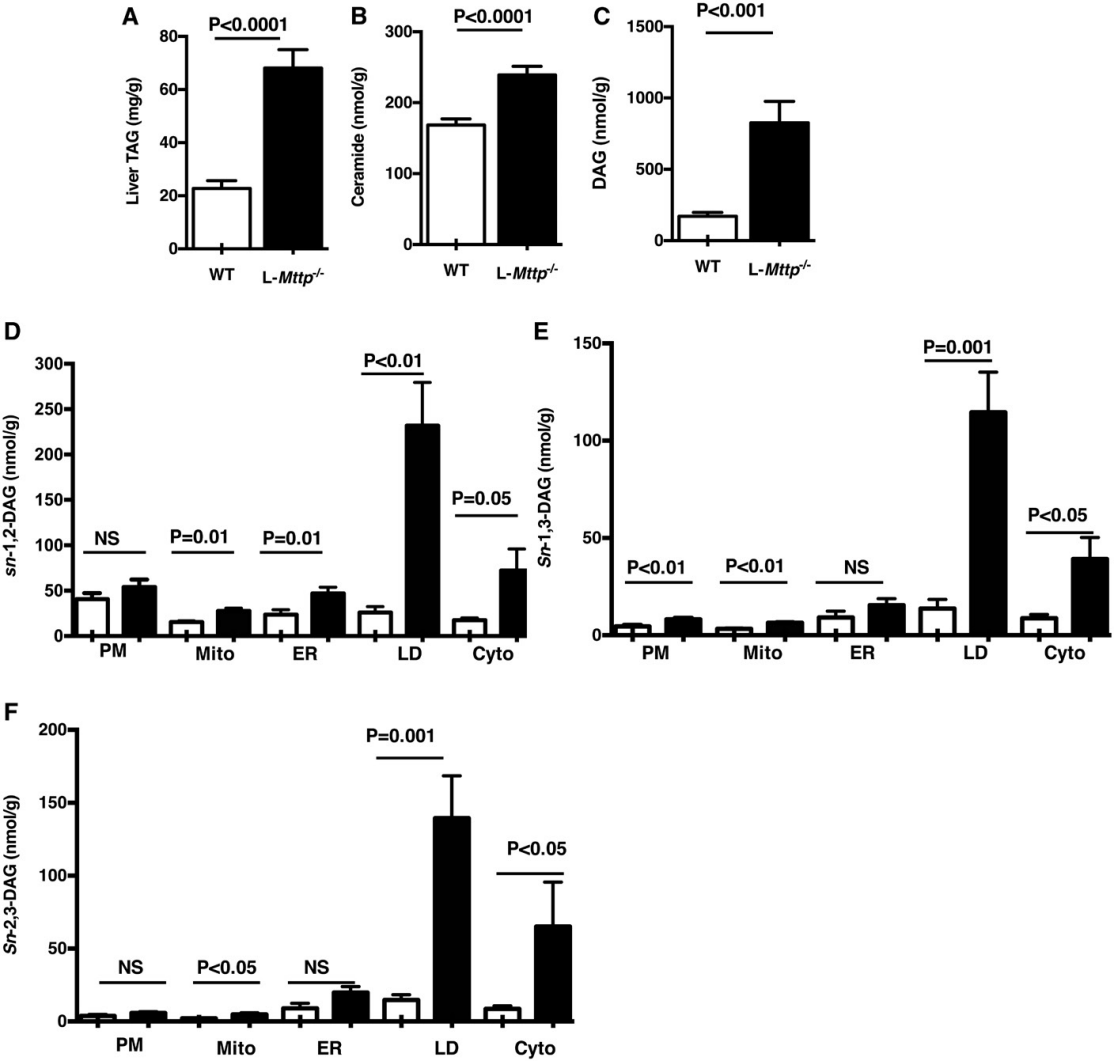
Young L-*Mttp*^−/−^ mice develop hepatic steatosis without the accumulation of plasma membrane *sn*-1,2-DAGs. A: Liver TAG content. B: Liver ceramide content. C: Total DAG. D–F: *sn*-1,2-DAG (D), *sn-*1,3-DAG (E), and *sn-*2,3-DAG (F) content in five compartments of the liver from young WT and L*-Mttp^−/−^* mice: plasma membrane (PM), mitochondrial (Mito), ER, lipid droplet (LD), and cytosol (Cyto). Individual statistical comparisons were evaluated by Student’s two-tailed *t*-test. Data are means ± SEMs of *n* = 6–8 per group.

Hepatic triglyceride was measured in 10–12-week-old L*-Mttp^−/−^* mice and *Mttp^f/f^* WT controls. As expected, hepatic TAG was significantly increased in young L*-Mttp^−/−^* mice (**Fig. 1A**). DAGs and ceramides are two bioactive lipid metabolites that are thought to link hepatic steatosis to hepatic insulin resistance. Hepatic ceramides and DAGs were both increased in young L*-Mttp^−/−^* mice (Fig. 1B, C). DAG accumulation causes hepatic insulin resistance through the activation of PKCε that results in phosphorylation of the insulin receptor threonine^11160^, which in turn leads to the inhibition of insulin receptor tyrosine kinase activity (10, 11, 26). It has recently been shown that *sn*-1,2-DAG at the plasma membrane is the entity that drives PKCε translocation and insulin resistance (27); thus, we measured *sn*-1,2-DAGs as well as the other DAG stereoisomers in five intracellular compartments. Using this approach, we found that *sn*-1,2-DAGs were increased in the lipid droplet, cytosol, ER, and mitochondrial compartments but not the plasma membrane compartment (Fig. 1D–F) of young L*-Mttp^−/−^* mice.

### Liver insulin sensitivity is preserved in young L-*Mttp*^−/−^ mice

**Fig. 2.**
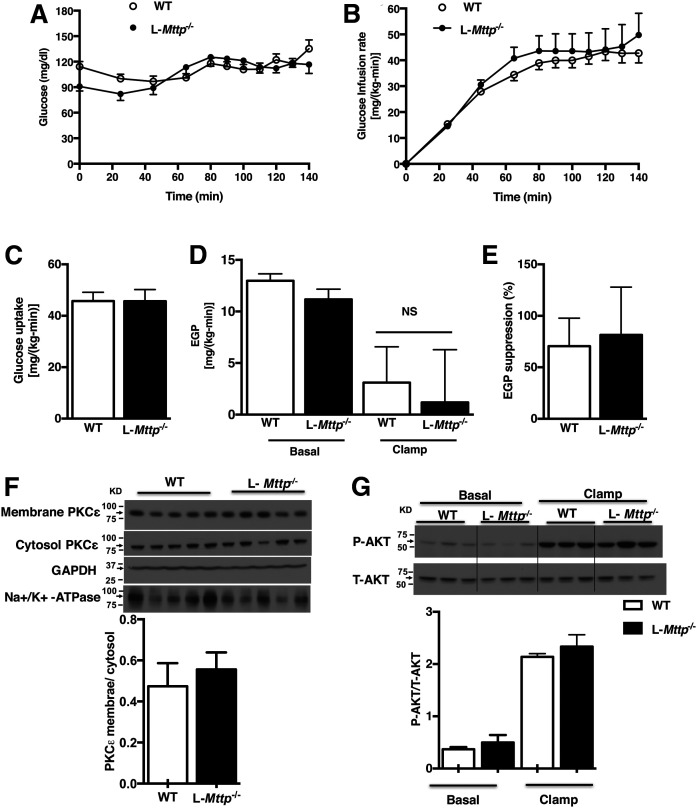
Normal whole-body insulin sensitivity in young L-*Mttp*^−/−^ mice. A, B: Plasma glucose concentrations (A) and glucose infusion rate (B) during the hyperinsulinemic portion of the clamp study. C: Whole-body insulin-stimulated peripheral glucose metabolism. D: Endogenous glucose production. E: Insulin-mediated suppression of endogenous glucose production. F: PKCε translocation in the liver. G: Liver AKT phosphorylation comparing basal and insulin-stimulated liver. Statistical comparisons were made by unpaired two-way Student’s *t*-test. Data are means ± SEMs of *n* = 7–8 per group.

We also assessed whole-body and tissue-specific insulin action by hyperinsulinemic-euglycemic clamp studies in 10–12-week-old L*-Mttp^−/−^* mice. Whole-body insulin sensitivity of young L*-Mttp^−/−,^* mice, as reflected by the glucose infusion rate required to maintain euglycemia, was not different compared with WT mice (**Fig. 2A**, B). Furthermore, insulin-stimulated peripheral glucose disposal was not changed (Fig. 2C). Additionally, insulin-mediated suppression of endogenous glucose production (EGP) was also not altered in young L*-Mttp^−/−^*mice, indicating unchanged hepatic insulin sensitivity (Fig. 2D, E). Thus, MTTP deficiency does not alter hepatic or peripheral insulin action in young mice despite marked hepatic steatosis and increases in *sn*-1,2-DAGs in the lipid droplet fraction.

PKCε activation links hepatic DAG accumulation to hepatic insulin resistance. Young L*-Mttp^−/−^* mice displayed no change in hepatic PKCε membrane translocation, a surrogate for PKCε activation, compared with WT mice (Fig. 2F), consistent with the unchanged *sn*-1,2-DAG in the hepatic plasma membrane. Furthermore, insulin-stimulated protein kinase B (Akt) phosphorylation was not changed, reflecting preserved hepatic insulin signaling in young L*-Mttp^−/−^* mice (Fig. 2G).

### Aged L*-Mttp*^−/−^ mice demonstrate CRMP-reversible glucose intolerance

**Fig. 3.**
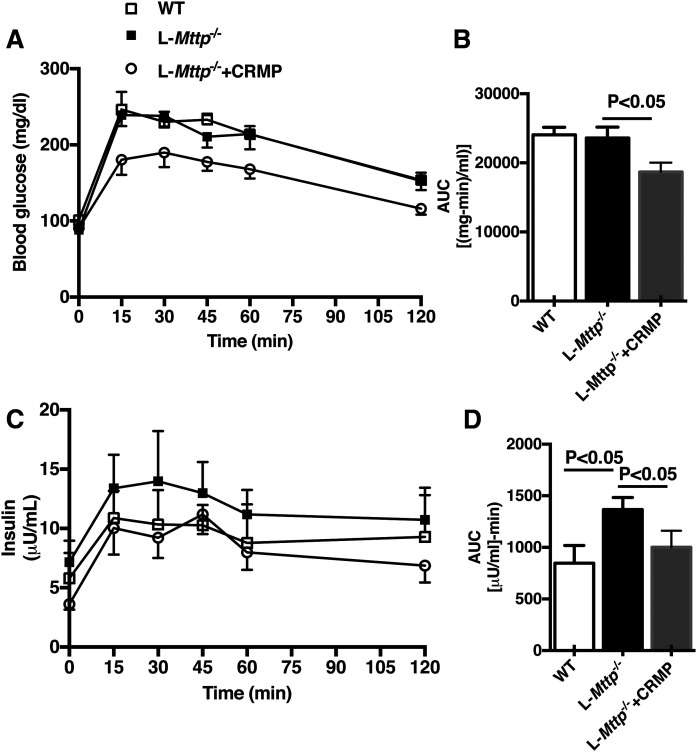
Aged L*-Mttp^−/−^* mice demonstrated glucose intolerance that improved with CRMP. A, B: Plasma glucose concentration time course (A) and area under the glucose versus time curve (B) during intraperitoneal glucose tolerance tests. C, D: Plasma insulin concentration time course (C) and area under the insulin versus time curve (D) during intraperitoneal glucose tolerance tests. Mice were fasted overnight before the glucose tolerance tests. Data are represented as means ± SEMs. Statistical comparisons were made by two-way ANOVA. Data are means ± SEMs of *n* = 7–8 per group.

We evaluated 10-month-old L*-Mttp^-/^* mice and WT controls to gauge the metabolic impact of the interaction between aging and hepatic Mttp deficiency. We first evaluated L*-Mttp^-/^* mice by intraperitoneal glucose tolerance testing. Although the excursion in plasma glucose concentration was not altered during glucose tolerance testing (**Fig. 3A**, B), the plasma insulin excursions were much higher in aged L*-Mttp^−/−^* mice. These changes in plasma insulin with normal plasma glucose concentrations are a more sensitive measure of hepatic insulin resistance during a glucose tolerance test, and these insulin excursions were higher in aged L*-Mttp^−/−^* mice (Fig. 3C, D). To determine whether insulin resistance could be reversed with a liver-targeted mitochondrial uncoupler to decrease liver fat content, we treated L-*Mttp^−/−^* mice with the controlled-release form of 2,4-dinitrophenol, CRMP. CRMP-treated L*-Mttp*^−/−^ mice were more glucose-tolerant than untreated L*-Mttp*^−/−^ mice, as reflected by 20%–30% reductions in plasma glucose and insulin concentrations during the intraperitoneal glucose tolerance test. (Fig. 3A–D).

### Hepatic steatosis and PKCε activation in aged L*-Mttp*^−/−^ mice are blunted by CRMP administration

**Fig. 4.**
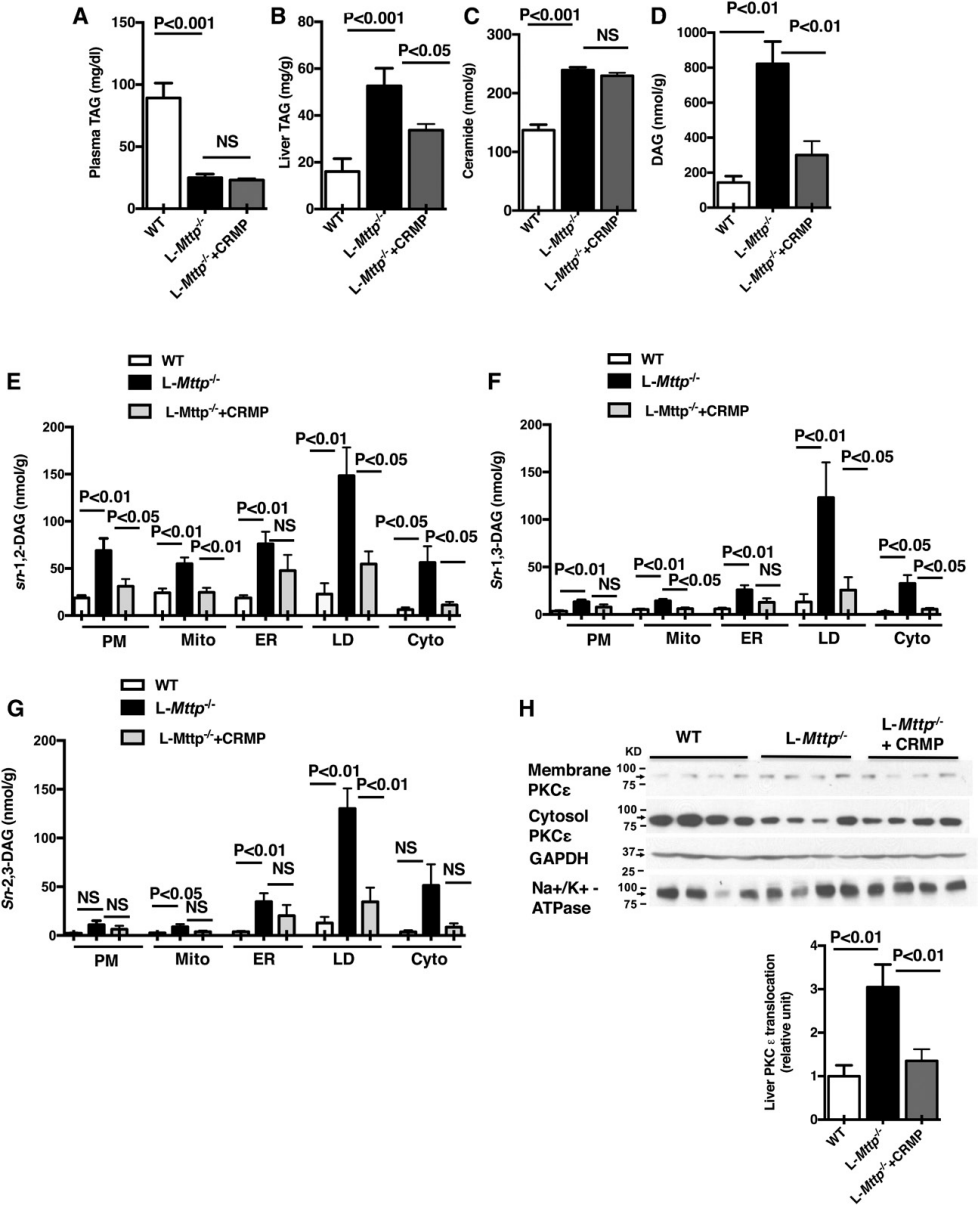
Hepatic steatosis, hepatic plasma membrane *sn*-1,2 DAG accumulation, and increased PKCε membrane translocation were all observed in aged L-*Mttp*^−/−^ mice. A: Plasma TAG concentration. B: Liver TAG content. C: Liver ceramide content. D: Liver total DAG. E–G: *sn*-1,2-DAG (E), *sn-*1,3-DAG (F), and *sn-*2,3-DAG (G) content in five hepatic subcellular compartments from aged WT mice, aged L*-Mttp^−/−^* mice, and aged L*-Mttp^−/−^* mice treated with CRMP: plasma membrane (PM), mitochondrial (Mito), ER, lipid droplet (LD), and cytosol (Cyto). H: Hepatic PKCε translocation. Statistical comparisons were made by two-way ANOVA. Data are means ± SEMs of *n* = 6–8 per group.

We evaluated plasma and hepatic lipid content in our aged WT, L*-Mttp*^−/−^, and CRMP-treated L*-Mttp*^−/−^ mice. Fasting plasma TAG was decreased by 70% in L*-Mttp*^−/−^ versus WT mice; this lower level of plasma TAG was unaffected by CRMP intervention (**Fig. 4A**). Hepatic TAG was increased in L*-Mttp*^−/−^ versus WT mice; 4 weeks of CRMP treatment decreased hepatic TAG in L*-Mttp*^−/−^ mice by 15% (Fig. 4B). Hepatic ceramide levels were increased in aged L*-Mttp^−/−^* mice; however, these levels were not changed by CRMP treatment (Fig. 4C). In contrast, total DAG was increased in aged L*-Mttp^−/−^* mice, and this increase was dramatically blunted with CRMP treatment (Fig. 4D). We assessed DAG stereoisomers in five subcellular compartments. In the aged L*-Mttp*^−/−^ mice, *sn*-1,2–DAG content was increased in all subcellular compartments, contrasting with the young L*-Mttp*^−/−^ mice. The increase in DAG was attenuated by CRMP treatment (Fig. 4E–G). PKCε membrane translocation was increased in aged L*-Mttp^−/−^* mice, and this increase in PKCε activation was reversed by CRMP treatment (Fig. 4H). Thus, CRMP treatment effectively reduced the accumulation of hepatic TAG and DAG content in aged L*-Mttp^−/−^* mice. Given our previous results it is likely that the decrease in plasma membrane *sn*-1,2-DAG accounts for the decrease in PKCε activation in these aged L*-Mttp^−/−^* mice (27).

### Whole-body energy balance was not altered by L*-Mttp^−/−^* genotype or CRMP treatment

**Fig. 5.**
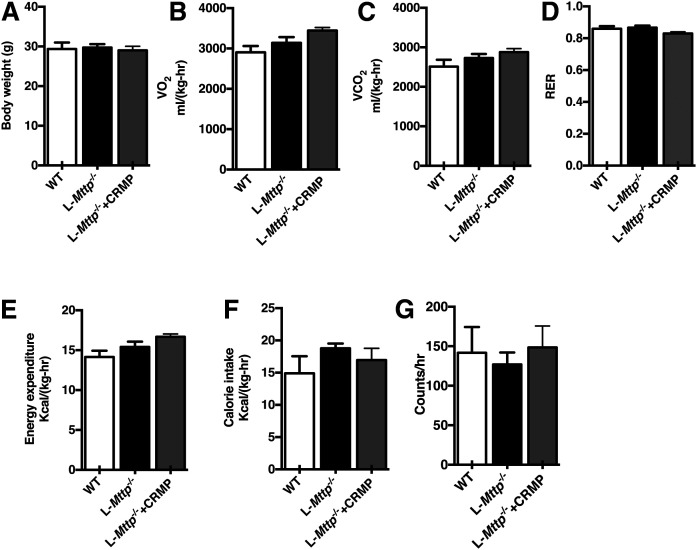
Whole-body energy balance was not different between aged WT mice, aged L*-Mttp^−/−^* mice, and aged L*-Mttp^−/−^* mice treated with CRMP. A: Body weight. B: Oxygen consumption (V_O2_). C: Carbon dioxide production (V_CO2_). D: Respiratory exchange ratio. E: Energy expenditure throughout the day. F: Food intake. G: Daily activity. Statistical comparisons were made by two-way ANOVA. Data are means ± SEMs of *n* = 6 per group.

To examine whether the deficiency of MTTP in the liver has an effect on whole-body energy balance, we performed metabolic cage studies with aged WT mice, aged L*-Mttp^−/−^* mice, and aged L*-Mttp^−/−^* mice treated with CRMP. Neither deficiency of MTTP nor CRMP treatment altered body weight (**Fig. 5A**). Consistent with the matched body weights, there were no differences in oxygen consumption, carbon dioxide production, respiratory quotient, energy expenditure, feeding, and activity during metabolic cage studies in any of the three groups (Fig. 5B–G). Thus, while we observed significant differences in glucose tolerance between aged WT and L-*Mttp*^−/−^ mice and between L-*Mttp*^−/−^ mice with and without CRMP treatment, these differences were not associated with changes in whole-body energy balance, body weight, or activity.

### Liver inflammation and ER stress were not changed in aged L-*Mttp^−/−^* mice

**Fig. 6.**
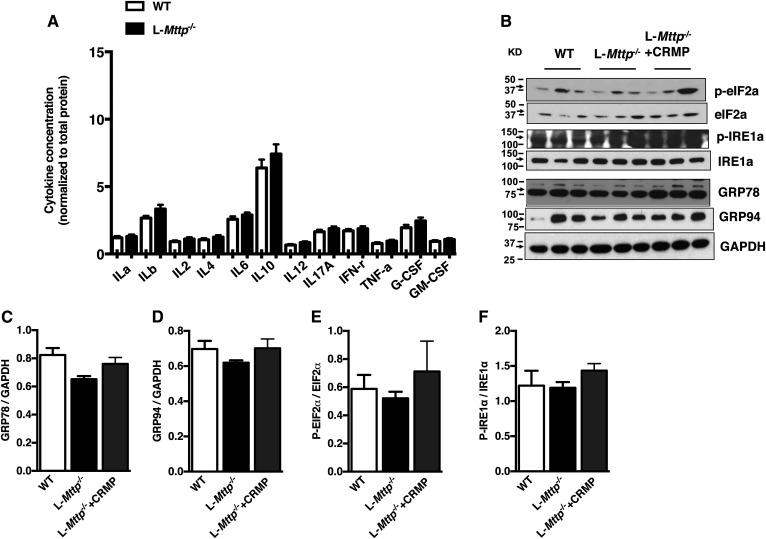
Liver inflammatory markers and activation of the unfolded protein response were unaltered by genotype or drug treatment in aged L*-Mttp^−/−^* mice and L*-Mttp^−/−^* mice treated with CRMP. A: Hepatic cytokine concentrations. B: ER stress/unfolded protein response markers assessed by immunoblot. C: GRP78. D: GRP94. E: Phosphorylation of eIF2. F: Phosphorylation of IRE1. Statistical comparisons were made by two-way ANOVA. Data are means ± SEMs of *n* = 3 per group.

Inflammation and ER stress are major factors that promote insulin resistance (28, 29). We assessed whether inflammatory changes were associated with the alterations in insulin action observed in aged L*-Mttp^−/−^* mice. Hepatic proinflammatory and antiinflammatory cytokine content was measured: IL1α, IL1β, IL2, IL4, IL6, IL10, and IL12 were not changed in aged L*-Mttp^−/−^* mice (**Fig. 6A**) compared with WT mice. We also assessed for any changes in ER stress that might also contribute to the observed changes in hepatic insulin action in aged L*-Mttp^−/−^* mice. ER stress markers were assessed by immunoblotting and were not altered in the liver tissue of aged L*-Mttp^-/^*mice and aged L*-Mttp^-/^*mice treated with CRMP (Fig. 6B–F). Thus, changes in liver inflammation and changes in ER stress do not appear to account for the changes in glucose tolerance we observed in aged L*-Mttp^−/−^* mice.

### CRMP reversed hepatic insulin resistance in aged L-*Mttp*^−/−^ mice

**Fig. 7.**
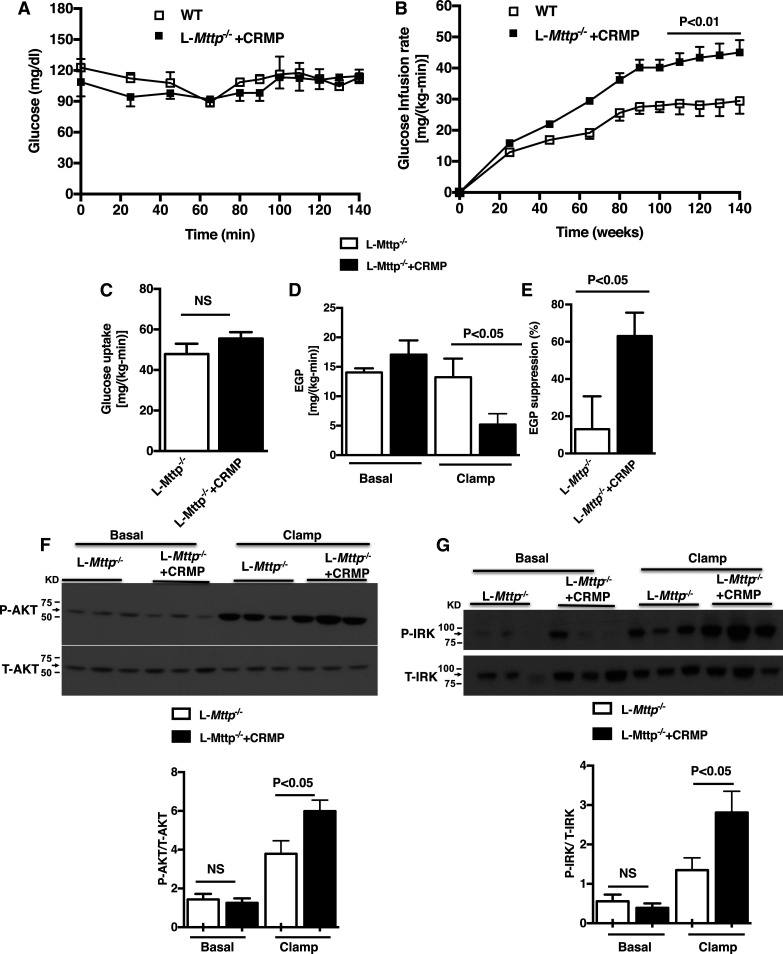
CRMP treatment improved hepatic insulin sensitivity of aged L*-Mttp^−/−^* mice as assessed by hyperinsulinemic-euglycemic clamps. A: Plasma glucose during the clamp. B: Glucose infusion rate during the clamp. C: Insulin-stimulated peripheral glucose metabolism. D: EGP in both the basal and hyperinsulinemic clamped state. E: Insulin-mediated suppression of EGP represented as percentage suppression. F: Liver AKT phosphorylation under basal and insulin-stimulated conditions. G: Liver insulin receptor β (insulin receptor kinase; IRK) phosphorylation under basal and insulin-stimulated conditions. Statistical comparisons made by Student’s *t*-test. Data are means ± SEMs of *n* = 6–7 per group.

Taken together, the observed differences in hepatic DAG content and hepatic PKCε activation suggest that the improvement in glucose tolerance in aged L*-Mttp^−/−^* mice treated with CRMP is due to a reversal in lipid-induced hepatic insulin resistance. To demonstrate this definitively, we performed hyperinsulinemic-euglycemic clamps on aged L*-Mttp^−/−^* mice treated for 4 weeks with CRMP or vehicle. CRMP-treated mice showed a significant improvement in whole-body insulin sensitivity, reflected by an increased glucose infusion rate during the clamp (Fig. 7A, B). Insulin-stimulated peripheral glucose disposal was not changed (Fig. 7C). Insulin-mediated suppression of EGP was significantly increased in CRMP-treated mice, indicating improved hepatic insulin responsiveness (Fig. 7D, E). Consistent with changes in hepatic insulin sensitivity, insulin-stimulated protein kinase B and insulin receptor kinase phosphorylation were significantly increased in the liver tissues of L*-Mttp^−/−^* mice treated with CRMP (Fig. 7F, G). Taken together these results demonstrate that CRMP’s ability to improve glucose tolerance in aged L*-Mttp*^−/−^ mice could mostly be attributed to the reversal of hepatic insulin resistance.

## DISCUSSION

MTTP inhibitors have been shown to reduce circulating VLDL and LDL in animal models and human subjects (2, 30, 31) and thus are a valuable addition to the pharmacopoeia for patients with severe hypertriglyceridemia. However, the promise of MTTP-targeted therapeutics has been questioned, as hepatic MTTP inhibition or MTTP deficiency induces hepatic steatosis and transaminitis (8, 32, 33). NAFLD is of great clinical concern, both as a precursor to nonalcoholic steatohepatitis and as a cause of hepatic insulin resistance (34). Prior investigations have observed that MTTP knockout mice develop hepatic steatosis, with increased DAG and ceramide content but without the development of hepatic insulin resistance or glucose intolerance (1). Furthermore, treating Zucker fatty rats with an MTTP inhibitor not only improved their glucose tolerance but also decreased fasting plasma glucose and insulin concentrations (9), consistent with a reversal of insulin resistance. Until now, there has been no explanation for the discordance between hepatic bioactive lipid content and hepatic insulin action in these models. Our study both recapitulates and explains these findings. We found that L*-Mttp^−/−^* mice develop hepatic steatosis at an early age but do not develop hepatic insulin resistance. However, we also found that with aging, L*-Mttp^−/−^* (∼10-month-old) mice do indeed develop hepatic insulin resistance. The discordant metabolic phenotypes of the young and aged L*-Mttp^−/−^* mice were explained well in the context of the DAG-PKCε hypothesis of lipid-induced hepatic insulin resistance (34, 35); the key to understanding the phenotype of these mice was an analysis of the DAG stereoisomer content separated by subcellular compartment. Both young and aged L*-Mttp^−/−^* mice had increased total hepatic DAG content, but only the aged mice demonstrated increases in plasma membrane *sn*-1,2-DAG and PKCε activation.

The DAG-nPKC model helps to explain lipid-induced insulin resistance in multiple tissues (34, 35). The results from this study support this hypothesis, wherein *sn*-1,2-DAG accumulation in the hepatic plasma membrane drive plasma membrane PKCε recruitment and activation. Activated PKCε in turn phosphorylates threonine^1160^ in the tyrosine kinase active site of the insulin receptor, destabilizing this site and disrupting insulin receptor tyrosine kinase activity (10, 11). Thus, in the setting of increased hepatic plasma membrane *sn*-1,2-DAG more insulin must bind the hepatic insulin receptor to drive the multiple downstream hepatocellular actions of insulin; that is, the liver is insulin-resistant. Of note, there are models that dissociate hepatic lipids from hepatic insulin resistance. Some, such as sobetirome (a thyroid hormone receptor-β agonist) treated rodents, demonstrate increased hepatic glucose production despite reduced hepatic lipids due to the increased flux of gluconeogenic precursors (36). Others, such as the CGI58 knockdown mouse, demonstrate increased hepatic DAG content but no increase in PKCε activation due to the storage of DAG in lipid droplets, which represent a neutral compartment (12, 37). The lack of hepatic insulin resistance in the L-*Mttp^−/−^* mouse appears to be consistent with this latter mechanism. Consistent with prior studies of the L-*Mttp^−/−^* mouse (1), in the young L-*Mttp^−/−^* mouse we observed increased hepatic bioactive lipid species without an increase in hepatic insulin resistance. To explain this discrepancy between hepatic lipid and hepatic insulin resistance, we fractionated the DAGs both by subcellular compartment and by stereochemistry, as PKCs are thought to be activated by plasma membrane *sn*-1,2-DAG (27, 38–40) and not by DAGs in other compartments or 1,3- or 2,3-DAG stereoisomers. The *sn*-1,2-DAG stereoisomer content was not increased in the plasma membrane from the livers of these mice, explaining the insulin-sensitive hepatic steatosis phenotype. Furthermore, in contrast with both prior studies of young L-*Mttp^−/−^* mice and our study of young L-*Mttp^−/−^* mice, in aged L-*Mttp^−/−^* mice we saw increased hepatic insulin resistance, along with a concomitant increase in plasma membrane *sn*-1,2-DAG content. These patterns correlating plasma membrane DAG content with hepatic insulin resistance were most clearly observed with *sn*-1,2-DAG stereoisomers. Thus, we found hepatic insulin resistance and PKCε activation was dissociated from hepatic steatosis and total TAG/DAG content in young L-*Mttp^−/−^* mice, as the *sn*-1,2-DAGs were stored in neutral compartments (mostly lipid droplets) in these mice, while hepatic insulin resistance developed in the older L-*Mttp^−/−^* mice as *sn*-1,2-DAGs accumulated in the plasma membrane, leading to PKCε activation and decreased hepatic insulin signaling at the level of the insulin receptor.

The discrepancy between the DAG localization and the phenotypes of the young versus old mice may provide insight into how DAGs are trafficked from the ER to the plasma membrane. Our results suggest that DAG transport from the ER to the plasma membrane may occur by multiple pathways. DAG would accumulate rapidly during VLDL secretion and more slowly by pathways independent of VLDL secretion. This model could be tested by application to other genetic variants associated with defective VLDL secretion. Mutations in APOB and TM6SF2 both lead to defective VLDL secretion and reduced LDL-C and are associated with human disease. APOB mutations cause familial hypobetalipoproteinemia, while TM6SF2 mutations are associated with NAFLD and progression to NASH (41–44). *Apob* mutant mice are quite phenotypically similar to MTTP-deficient mice, developing hepatic steatosis (41) without a disruption in glucose or insulin tolerance (42). While the protection against insulin resistance seen in ApoB mice may be due to a reduction in the delivery of TAG to the muscle and reduced peripheral insulin resistance, it would be reasonable to speculate that animals with defective VLDL secretion may be slower to develop hepatic insulin resistance due to a reduction in DAG transportation from the ER to the plasma membrane. The assessment of hepatic insulin action and plasma membrane DAG content in young and older ApoB and Tm6sf2 mutant mice would help to test a multipathway model of DAG transport from ER to plasma membrane.

The MTTP inhibitor lomitapide has been approved to treat patients with homozygous familial hypercholesterolemia; however, this approach induces hepatic fat accumulation and increased plasma transaminases (5, 7, 45). The use of lomitapide is associated with increased circulating transaminases, and this medication carries a boxed warning regarding hepatotoxicity. If the hepatic fat accumulation associated with MTTP inhibition can be prevented, the hepatotoxicity of the drug class may be attenuated. In this study, we examined the effect of a functionally liver-targeted mitochondrial protonophore, CRMP (14), in aged L-*Mttp*^−/−^ mice. We found that CRMP treatment reversed hepatic fat accumulation and improved glucose tolerance in aged L-*Mttp*^−/−^ mice. Consistent with these improvements in hepatic steatosis and in glucose tolerance, CRMP treatment markedly increased both whole-body insulin responsiveness and hepatic insulin sensitivity. The improvements in hepatic insulin sensitivity were associated with reductions in plasma membrane *sn*-1,2-DAG content, PKCε activation, and normalized insulin signaling from the insulin receptor through Akt in aged L-*Mttp*^−/−^ mice. In contrast, there were no changes in hepatic ceramide content despite the reversal of hepatic insulin resistance with CRMP treatment. Thus, while liver-specific aged L-*Mttp*^−/−^ mice developed hepatic insulin resistance, this defect in hepatic insulin action was reversible by CRMP treatment. These findings offer further evidence for the role of plasma membrane *sn*-1,2-DAG -induced PKCε activation in causing hepatic insulin resistance and support the potential utility of CRMP and other liver-targeted mitochondrial protonophores for the treatment of NAFLD associated with reduced MTTP activity. These results also dissociate hepatic ceramide content from hepatic insulin resistance in these CRMP-treated L-*Mttp*^−/−^ mice, demonstrating that ceramides are involved in causing hepatic insulin resistance in this model.

Taken together, these data demonstrate that differences in the intracellular compartmentation of *sn*-1,2-DAGs in the lipid droplet versus plasma membrane explain the dissociation of NAFLD and lipid-induced hepatic insulin resistance in young L-*Mttp*^−/−^ mice as well as the development of lipid-induced hepatic insulin resistance in aged L-*Mttp*^−/−^ mice. Consistent with the key role for plasma membrane *sn*-1,2-DAG-induced PKCε activation in causing hepatic insulin resistance in the aged L-*Mttp*^−/−^ mice, we show that liver-targeted mitochondrial uncoupling with CRMP reverses hepatic steatosis, plasma membrane *sn*-1,2-DAG accumulation, hepatic PKCε activation, and hepatic insulin resistance in these mice.

### Data availability

All data used are contained within the article.
